# Suzetrigine: a novel non-opioid for acute postoperative pasin relief

**DOI:** 10.1097/MS9.0000000000004229

**Published:** 2025-11-21

**Authors:** Ume Aiman, Vishan Das, Muhammad Salar, Haseeb Safdar Ali, Muddassir Khalid

**Affiliations:** a Department of Medicine, Liaquat University of Medical and Health Sciences, Jamshoro, Pakistan; b Department of Medicine, Chandka Medical College, Larkana, Sindh, Pakistan; c Department of Medicine, Nishtar Medical University, Multan, Pakistan

*Dear Editor,*

Postoperative pain remains insufficiently controlled in a considerable number of patients. It is linked to a broad range of adverse outcomes, including higher morbidity, chronic postoperative pain, impaired function, recovery from surgery, reduced quality of life, prolonged opioid use, and increased health care costs^[1]^. Persistent pain alters gene expression of pain-modulating cells, sustaining a pronociceptive state. The overexpression of VGSC increases the excitability of neurons that sense pain^[2]^.

Postoperative pain continues to be one of the most important clinical concerns in postoperative care, influencing the length of recovery, quality of life, and risk of prolonged use of opioids. In spite of the availability of acetaminophen, NSAIDs, and regional anesthetic techniques, opioids remain the most common and often improperly prescribed postoperative pain management. With the approval of the first-in-class selective NaV1.8 inhibitor suzetrigine in 2025, we now have a novel non-opioid option for the management of acute postoperative pain. Common non-opioid side effects may restrict their use as opioid-sparing analgesics and need cautious prescribing. On the contrary, the short duration of regional analgesia frequently limits its effectiveness. Evidence suggests that opioids are commonly prescribed in excess following surgery, with likely inadequate follow-up. According to postsurgical statistics, these reports raise concern due to the reported frequency of persistent postoperative opioid use^[3]^.

Suzetrigine was developed in response to the significant unmet demand for an effective pharmacological non-opioid drug to treat acute pain without running the risk of addiction and physical dependence. Since suzetrigine is a non-opioid selective NaV1.8 inhibitor, it is not susceptible to the negative effects of opioids. According to gene expression data, NaV1.8 is not present in any of the 190 different parts of the human brain or spinal cord, preventing harmful CNS effects. It exhibits the phenomenon of “use dependence” and has the qualities of rapid absorption. One hour following oral treatment, the peak plasma concentration was reached with 71% oral bioavailability. Both intravenous and oral administration had similar half-lives (5.9 hours)^[4]^.

The effectiveness of investigational analgesics for a wide range of acute pain is commonly evaluated using abdominoplasty and bunionectomy, two well-known clinical models for assessing soft tissue and hard tissue (bone) pain, respectively. Suzetrigine monotherapy was found to be safe and well tolerated in two previous phase 2 trials, and it resulted in statistically significant and clinically relevant decreases in moderate-to-severe acute pain after abdominoplasty or bunionectomy when compared with a placebo. In the abdominoplasty trial, 68% of suzetrigine-treated patients reported good or excellent pain control at 48 hours, compared to 50% of placebo-treated patients (*P* < 0.001). Compared to 40% of patients in the placebo group, 53% of patients in the suzetrigine group reported good or excellent pain control during the bunionectomy trial (*P* = 0.034)^[5]^. In conclusion, Suzetrigine is a selective Nav1.8 inhibitor and the first drug of its kind to be approved by the FDA for the treatment of acute pain. It is a peripheral sodium channel inhibitor with low penetration into the CNS and has been found to lack sedation, euphoria, and respiratory depression that are commonly observed with opioids, while still providing clinically meaningful analgesia.

TITAN Guidelines: This manuscript is in compliant to TITAN Guidelines, 2025, declaring no use of AI^[6]^.

## Data Availability

Not applicable.
